# Untangling the Genetics of Human Longevity—A Challenging Quest

**DOI:** 10.3390/genes10080585

**Published:** 2019-07-31

**Authors:** Serena Dato, Mette Soerensen, Giuseppina Rose

**Affiliations:** 1 Department of Biology, Ecology and Earth Sciences, University of Calabria, 87036 Rende, Italy; 2 The Danish Twin Registry and Epidemiology, Biodemography and Biostatistics, Department of Public Health, University of Southern Denmark, 5000 Odense-C, Denmark

**Keywords:** genetic determinants of human longevity, genetic variation, genetic association study, longevity-related genes, human lifespan

Human average life expectancy in developed countries has increased dramatically in the last century, a phenomenon which is potentially accompanied by a significant rise in multi-morbidity and frailty among older individuals. Nevertheless, some individuals appear someway resistant to causes of death, such as cancer and heart disease, compared with the rest of the population, and are able to reach very old ages in good clinical conditions, while others are not. Thus, during the last two decades we have witnessed an increase in the number of studies on biological and molecular factors associated with the variation in healthy aging and longevity. Several lines of evidence support the genetic basis of longevity: from the species-specific maximum lifespan to the genetically determined premature aging syndromes. Studies in human twins, that aimed to distinguish the genetic from the environmental component, highlighted a heritability of life span close to 25%. In centenarian’s families, the offspring of long-lived individuals not only exhibit a survival advantage compared to their peers, but also have a lower incidence of age-related diseases. On the other hand, population studies found that genetic factors influence longevity in age- and sex-specific ways, with a most pronounced effect at advanced age and possibly in men compared to women. All this evidence indicates that a genetic influence on longevity exists, laying the foundation for the search for the genetic components of extreme long life. Consequently, over the past three decades, there has been a surge in genetic research, due in part to advances in molecular technologies, starting as studies of single genetic variants in candidate genes and pathways, moving on to array-based genome-wide association studies (GWAS) and subsequently to next generation sequencing (NGS). However, despite a plethora of studies, only few variants (in the *APOE*, *FOXO3A* and *5q33.3* loci) have been successfully replicated in different ethnic groups and the emerging picture is complex. For instance, it is an understatement to think that long-lived people harbor only favorable variants, completely avoiding risk alleles for major age-related diseases; indeed, there is evidence that many disease alleles are present in long-lived people. It is more probable that the longevity phenotype is the result of a particular combination of pro-longevity variants and risk alleles for pathologies, likely interacting in networks in a sex- and age-specific way. Finally, characteristics of aging are extremely heterogeneous, even among long-lived individuals, due to the complex interaction among genetic factors, environment, lifestyle, culture and resiliency. Population and study specificity, lack of statistical power for such a rather rare phenotype and missing heritability represent further hard obstacles to overcome in genotype–phenotype association studies. Thus, many challenges remain to be addressed in the search for the genetic components of human longevity. In this Special Issue we included five original articles and two reviews covering different areas in the field of human longevity, to help the reader take stock of the situation and point to future perspectives of the field.

The two reviews in particular look closely at two main arguments of biogerontology research. The paper by Taormina and co-workers gives an updated review of the lessons from model organisms, where a substantial number of findings suggest that longevity could “directly” be promoted by interventions in specific pathways, like inflammation, oxidative stress response, DNA repair, as well as the use of nutrients [1]. The most relevant model systems used in biogerontology are discussed, as well as significant discoveries confirmed in humans, advising the researchers to use different model systems to avoid misinterpretation of the results due to confounding factors or model system peculiarities. The paper by Abondio et al. [2] reviews the available literature on *APOE* and its involvement in biological pathways related to human longevity, under an anthropological and population genetics perspective, highlighting the evolutionary dynamics, which may have shaped the distribution of its haplotypes across the globe and the potential adaptive role. Both of the two reviews are useful compendia of reference papers in the field and, at the same time, provide good points of discussion for future studies on the genetic aspects of human longevity.

The paper by Hjelmborg et al. discusses an interesting topic for demographical studies on human longevity, i.e., the role of zygosity in a twin’s lifespan [3]. Twin cohorts have been analyzed in several GWAS of common traits and diseases around the world. Although there is no evidence that the gene–disease associations seen in singletons differ in twins, the question if selecting one individual from a twin pair implies a selection in survival due to zygosity is still often questioned. The authors compared the relative survival of monozygotic (MZ) with dizygotic (DZ) twins (from the 1870–1900 and the 1961–1990 birth cohorts), from one of the largest nationwide cohorts of twins with valid vital status, the Danish Twin Registry; they found no correlation of mortality with zygosity, meaning that MZ and DZ pairs appear to share the same mortality process. Thus, being a twin does not appear to impact the basic biological processes and human development in adolescence and adulthood. This is an interesting result for the studies on disease onset and other age-dependent traits which use twins, because it implies that findings from twins are generalizable to the population as a whole, especially when large sample sizes are used.

The three candidate gene association studies of longevity presented here (the papers by Scarabino et al., De Rango et al. and Crocco et al.) directly deal with the search for the genetic component of longevity and healthy aging. The response to external injuries is the leitmotif unifying all three association studies.

As it is known, internal and external stresses disrupt telomere homeostasis. The contribution by Scarabino and co-workers confirms the genetic determination of leukocyte telomere length (LTL) by *TERT* variability, showing that shorter LTL at baseline may predict a shorter lifespan [4]. Furthermore, they found that the reliability of LTL as a lifespan biomarker could be age-specific and act in specific age-spans (age 70–79 in their study population).

The papers by De Rango et al. and Crocco et al. highlight the complexity of longevity, a highly dynamic phenotype influenced by internal and external stresses, that makes the identification of genes robustly associated with it very challenging. De Rango’s paper investigated for the first time the contribution to the longevity phenotype of the genetic variability of *IPMK* (Inositol Polyphosphate Multikinase), a potential moonlighting protein performing multiple functions in pathways affecting the aging process, from nutrient-sensing to oxidative stress and telomere maintenance [5]. This paper supports this gene as a novel gender-specific determinant of human longevity on one hand, and on the other hand promotes pleiotropic proteins like IPMK, able to integrate cellular activities in space and time, as crucial determinants of the complex connections among aging, health, and longevity. Dynamic genetic effects on longevity were found in the paper by Crocco et al. who investigated the variability of xenobiotic metabolizing genes, known to mediate the response/toxicity to xenobiotics [6]. They found lifelong changes in the frequency of alleles at *CYP2B6*, *CYP3A5*, *COMT* and *ABCC2* genes, following either linear or non-linear trajectories with respect to the chance of becoming long-lived. Such findings underline once again that SNPs associated with longevity might behave either as pro-longevity or killing variants but also as deleterious variants neutralized by the protective effect of pro-longevity genes (buffered variant), an important aspect to take into account in disentangling the genetic contributors to human longevity.

Finally, the paper by Revelas and co-workers contributes to the open debate of whether extreme longevity is coupled to risks for major diseases [7]. The authors built a polygenic risk score for cardiovascular health, based on GWAS variants, cardiovascular-related risk factors (such as cholesterol levels) and cardiovascular multi-morbidity disease (myocardial infarction, coronary artery disease, stroke etc.). By exploring the genetic profiles of 95+ year old individuals, the authors found that these extremely long-lived subjects did not have lower polygenic risk for cardiovascular health as compared to younger subjects, thus supporting the theory that exceptional longevity does not necessarily imply the absence of risk factors for major age-related traits.

Overall, we believe these papers, highlighting different facets and the complexity of the studies on the genetics of human longevity, may help to understand the path research has taken in the field up to now, and to explore some possible interventions, taking advantage of the pathways highlighted and of the perspectives they are unveiling for the future.

So, where should the longevity genetic field go from here? Despite decades of genetic studies, the variants consistently identified as associated to human longevity explain only a small part of the estimated heritability. For facing the challenge of ‘missing heritability’, new and innovative approaches are needed. First of all, more studies are needed to elucidate the effect of rare variants with larger effect sizes, not captured by standard GWAS. The opportunity of high-throughput methodologies like NGS, together with large multi-center collaborative studies of extremely long-lived cohorts, can contribute to pave the way for untangling the networks involved in human longevity. In this sense, the study of epistatic effects of different genetic markers in gene-set and pathway-enrichment analyses, as well as the integration of several layers of biological variation (SNP, Copy Number Variants, epigenetic markers) in polygenic risk scores could further help too. The application of a more functional genomic approach like the collection of whole-exome sequencing, genome-wide epigenetic, cell-specific transcriptomic data and the integration of all these layers of genomic information can help to disentangle the determinants of lifespan. Finally, the collection of life-long environmental and lifestyle variables known to influence an individual’s health (like microbiome and nutrition), can significantly improve our chance to untangle the intricate interplay between genes and environment in determining the longevity phenotype.

## References

[1] Taormina G., Ferrante F., Vieni S., Grassi N., Russo A., Mirisola M.G. (2019). Longevity: Lesson from Model Organisms. Genes.

[2] Abondio P., Sazzini M., Garagnani P., Boattini A., Monti D., Franceschi C., Luiselli D., Giuliani C. (2019). The Genetic Variability of *APOE* in Different Human Populations and Its Implications for Longevity. Genes.

[3] Hjelmborg J., Larsen P., Kaprio J., McGue M., Scheike T., Hougaard P., Christensen K. (2019). Lifespans of Twins: Does Zygosity Matter?. Genes.

[4] Scarabino D., Peconi M., Pelliccia F., Corbo R.M. (2019). Analysis of the Association Between *TERC* and *TERT* Genetic Variation and Leukocyte Telomere Length and Human Lifespan-A Follow-Up Study. Genes.

[5] De Rango F., Crocco P., Iannone F., Saiardi A., Passarino G., Dato S., Rose G. (2019). Inositol Polyphosphate Multikinase (*IPMK*), a Gene Coding for a Potential Moonlighting Protein, Contributes to Human Female Longevity. Genes.

[6] Crocco P., Montesanto A., Dato S., Geracitano S., Iannone F., Passarino G., Rose G. (2019). Inter-Individual Variability in Xenobiotic-Metabolizing Enzymes: Implications for Human Aging and Longevity. Genes.

[7] Revelas M., Thalamuthu A., Oldmeadow C., Evans T.J., Armstrong N.J., Riveros C., Kwok J.B., Schofield P.R., Brodaty H., Scott R.J. (2019). Exceptional Longevity and Polygenic Risk for Cardiovascular Health. Genes.

